# Using Three Variable State Equations to Gain Insight into PEM Fuel Cell Membrane Thermodynamics

**DOI:** 10.1021/acsomega.5c03544

**Published:** 2025-07-19

**Authors:** Nicholas A. Ingarra, Krzysztof (Chris) J. Kobus

**Affiliations:** Department of Mechanical Engineering, 6918 Oakland University, Rochester Hills, Michigan 48309, United States

## Abstract

A PEM fuel cell is an electrochemical system dependent on multiple variables such as temperature, voltage, current, pressure, volume, and chemical potential. Thermodynamically, parts of this system are seen through simpler mechanical equations of state developed for subsets of the more complex system (a simple compressible substance, for instance), providing a piecewise understanding of the whole. In this research, a method using equations of state with three variables is proposed to arrive at the thermodynamic relationships in a more comprehensive way. As a result of three-variable equations of state, four additional equations of convenience are gained to complement enthalpy, Gibbs, and Helmholtz functions, providing additional relationships that contain a mix of mechanical and electrical physical quantities. The proposed approach is a more comprehensive path of gaining insight into a closer thermodynamic representation of a functioning fuel cell to in turn improve the understanding and modeling of this important electrochemical system.

## Graphical abstract

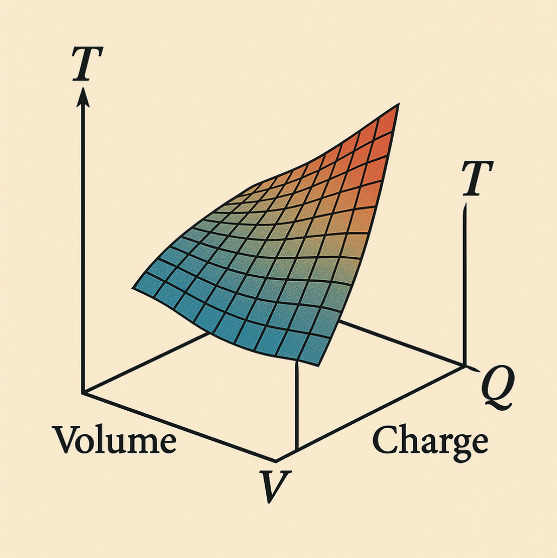

## Introduction

The current process of modeling a PEM fuel cell, at least thermodynamically, uses two-variable equations of state that are based on simpler processes.^1^ For instance, the isentropic state relationship for internal energy of a simple compressible substance is dependent on entropy, temperature, pressure, and volume:

du=Tds−pdv 1

Here the only work present is mechanical work (boundary work). There is no electrical work as it is a simple substance. With this simple differential equation of state as a starting point, other state equations are obtained such as enthalpy, h, Gibbs free energy, g, and the Helmholtz function, A, through the Legendre transform^1^ resulting in secondary differential equations of state:

dh=Tds+vdp 2

dg=−SdT+vdp 3

dA=−SdT−pdv 4

The principle differential equation of state, eq 1, along with these secondary ones have been utilized using Maxwell’s equations to yield equations of state relating nonmeasurable properties to measurable ones. To include electric work, conventionally it has been simply added to the above equations after the fact;^1–6^ thus, eqs 1 and 3, respectively, become:

$$du=Tds-pdv-{dW}_{e}$$ 5

$$dg=-sdT+vdp-{dW}_{e}$$ 6

If temperature and pressure remain constant, then the Gibbs free energy is equal to electric work which in turn is obtained from a closed system analysis with constant pressure and temperature;^1–6^ therefore reversibility is equal to the change in Gibbs free energy in that case. The change in Gibbs free energy is then equal to electric work for the isobaric and isothermal case:

$$dg=-{dW}_{e}$$ 7

The electrical work can also be written in terms of the chemical reaction, where

$$W_{e}=-\Delta g_{rxn}$$ 8

Further, electric work itself can be quantified in terms of voltage and charge, and using Faraday’s model results in

$$dg=W_{e}=\phi q=nF\phi$$ 9

If the electrical work from eq 8 is set equal to electrical work in eq 9, the theoretical voltage can be solved for; thus:

$$\phi^{o}=\frac{-\Delta g_{rxn}}{nF}$$ 10

It is noted that this theoretical voltage is based specifically on isobaric and isothermal conditions. The differential electrical work can also be expressed in terms of voltage and charge as

$$dg=dW_{e}=\phi dq$$ 11

The Gibbs free energy equation is based on isothermal and isobaric conditions and if these conditions are not met, corrections are needed. The first step in this case involves correcting for temperatures when the system is not at exactly the 25 °C reference temperature. The change in Gibbs free energy, eq 3, with respect to temperature is

$${\left(\frac{dG}{dT}\right)}_{p}=-S$$ 12

A results of the Gibbs free energy to entropy using eq 10 also yields

$${\left(\frac{\mathrm{d\Delta}g}{dT}\right)}_{p}=-\Delta S$$ 13

If the Gibbs free energy, eq 9, is differentiated with respect to temperature and set equal to the change in entropy from eq 13, then a relationship between voltage and entropy is obtained;^1,2^ thus,

$$\frac{\left(\Delta s\right)}{nF}=-\frac{d\phi }{dT}$$ 14

The current process involves the assumption of entropy being independent of temperature.^1^ If more accuracy is required, the specific heat capacities need to be integrated over the temperature range. Fuller and Harb^2^ noted that if the entropy change is not significant, then the entropy change over the temperature range, eq 14, is applicable. Like,^1^ Fuller and Harb^2^ stated that if the entropy change over the temperature range is significant then the entropy will need to be integrated over the temperature range and this will involve the specific heat. If constant pressure is assumed the voltage change with respect to temperature is

$$\phi_{T}=\phi^{o}+\frac{\Delta s}{\mathrm{nF}}\left(T-T_{o}\right)$$ 15

A similar process can be applied evaluating the impact of pressure on voltage. The first step involves using the equation of state of Gibbs free energy and taking the derivative of eq 3 with respect to pressure:

$${\left(\frac{dG}{dp}\right)}_{T}=v$$ 16

The change in Gibbs free energy with respect to pressure can be related to the change in volume by differentiating eq 3; thus,

$${\left(\frac{\mathrm{d\Delta}g}{\Delta p}\right)}_{T}=\Delta v$$ 17

If eq 10 is rearranged and differentiated with respect to pressure and set equal to the Gibbs relationship in eq 17, a new relationship is obtained:

$${\left(\frac{d\phi }{dP}\right)}_{T}=\frac{\Delta v}{nF}$$ 18

The current two variable method to calculate the impact of voltage on pressure involves substituting the ideal gas equation into eq 18 and solving the differential eq 1; thus,

$$\phi_{p}=\phi^{o}+\frac{RT}{nF}\ln\left(\frac{P_{water}P_{oxygen}^{1/2}}{P_{hydrogen}}\right)$$ 19

If on the other hand the electrical work in terms of voltage and current is added to the simple compressible substance and treated as a third variable, a new, more comprehensive, equation of state is obtained at the expense of the substance no longer being simple:

du=Tds−pdv+ϕdq 20

This introduces a mathematical complexity absent in the two-variable state equation, as Maxwell’s partial derivative relationships are no longer satisfied due to the inclusion of additional mixed partial derivatives. Consequently, a methodology is required where Maxwell’s mixed partial derivative relationships can still be applied, enabling the derivation of useful state equations that allow, for instance, voltage to be expressed as a function of mechanical properties such as pressure and temperature. The method used here to analyze a three-variable thermodynamic system is a superposition one first developed by Cai^7^ and Lungu.^8^ In a two-variable system there are only two mixed partials resulting from one of the tests for exactness. With the addition of a third variable, the possible mixed partial sets increase to eight. To resolve the issue, Cai^7^ and Lungu^8^ introduced a superposition method to still work with two variable sets at a time allowing Maxwell’s relationships to be used still with the end goal of getting nonmeasurable properties in terms measurable ones.

Cai^7^ focused on a substance with temperature, mechanical work and chemical potential, while Lungu^8^ on electricity and magnetism with some mechanical work. Their method of superposition of three-variable systems resulted in four additional equations of state that were not seen when 2-variable equations were combined from separate simpler analyses, this yielding additional relationships between thermodynamic properties. Some of the new relationships Lungu derived were the piezo-electric and pyro-magnetic coefficients. The Cai^7^ and Lungu^8^ methodology will be applied here specifically to variables associated with PEM fuel cells. The goal of this research, then, is to show how considering additional terms in fundamental state equations can serve to give rise to additional thermodynamic relationships, many of which are difficult to foresee. In the case of two variable systems, four equations are obtained (2^2^), but in the case of three variables the yield is eight (2^3^). The value of this is in the obtaining of these additional relationships that can then be used in analysis and modeling.

## Theory

A more comprehensive equation of state for an electrochemical system contains at least three terms rather than two, including mechanical as well as electrical properties as those are appropriate for consideration in a PEM fuel cell. For an entropy link to voltage, for instance, it would happen under isobaric and isothermal conditions. The conventional two-variable process in standard textbooks does not fully couple voltage, current, and charge to entropy, temperature, volume, and pressure. Assumptions are made to couple the mechanical properties to electrical ones by analyzing simpler sets of systems. There are a number of other properties that need to be taken into consideration for the case of a fuel cell such as chemical potential, electric, stress, and/or magnetic potential that considerably complicate the principal state eqs[Disp-formula eq8] and 9:

$$du=Tds-pdv+\epsilon dP+dW_{\epsilon }+\mu dN+\sigma d\epsilon_{m}+HdM+\varphi dq$$ 21

Equation 21 of course lays the groundwork for a far more complex analysis than eq 1 or for that matter eq 20. The question is then what relevant thermodynamic properties need to be retained to perform such a thermodynamic state analysis specifically regarding a PEM fuel cell. Cai^7^ and Lungu^8^ demonstrated the ability to work with 3 terms rather than 2. For instance, the Cai^7^ analysis resulted in a number of end thermodynamic relationships that were not born out of a combination of several 2-term analyses. Indeed, it appears that piecing together a number of 2-term systems to get a general understanding of more complex ones may miss some important information. A nonsimple dialectical substance has relevant thermodynamic properties such as entropy, pressure and volume (from boundary work), dipole moment and chemical potential;^8,9^ thus,

$$du=Tds-pdv+\epsilon dP+dW_{\epsilon }+\mu dN$$ 22

Focusing on the chemical-mechanical subsystem only, it would not contain any work from the movement of charged particles, simplifying eq 22:

du=Tds−pdv+μdN 23

Lungu^8^ examined the internal energy as a function of entropy, strain, dipole moment and magnetic field energy:

$$du=Tds+\sigma d\epsilon_{m}+EdP+HdM$$ 24

Lungu^8^ did not address the electrical work that is shown in eqs 11 and 20. A solution is needed to address entropy, temperature, pressure, volume, voltage and charge. The next step involves using three-variable thermodynamics shown in eq 20 to obtain equations of state in terms of measurable variables, which is the end goal. The entropy term is not directly measurable, so the relationships are obtained with mixed partials using superposition. The first superposition of eq 20 takes the first and second term equivalent to the two-variable case, and is purely mechanical:

$${\left(\frac{\partial T}{\partial v}\right)}_{s,q}=-{\left(\frac{\partial p}{\partial s}\right)}_{v,q}$$ 25

The second superposition involves the first and third terms of eq 20:

$${\left(\frac{\partial T}{\partial q}\right)}_{s,v}={\left(\frac{\partial \phi }{\partial s}\right)}_{q,v}$$ 26

This equation is the first coupling of entropy and electric properties. Equation 26 is not readily available in the archival literature.^2–5^ The last superposition is between the second and third terms of eq 20:

$$-{\left(\frac{\partial p}{\partial q}\right)}_{s,v}={\left(\frac{\partial \phi }{\partial v}\right)}_{s,q}$$ 27

Like the prior case, eq 27 is also not readily available in the archival literature. This is one of the positive features of coupling mechanical and electrical properties right from the principal differential equation of state. The next step involves creating other equations, which are done through the Legendre transform beginning with the secondary differential equation of state for enthalpy:

dh=Tds+vdp+ϕdq 28

Maxwell’s mixed partials are then applied beginning with the first and second terms, then first and third, and finally second and third terms resulting in

$${\left(\frac{\partial T}{\partial p}\right)}_{s,q}={\left(\frac{\partial v}{\partial s}\right)}_{p,q}$$ 29

$${\left(\frac{\partial T}{\partial q}\right)}_{s,p}={\left(\frac{\partial \phi }{\partial s}\right)}_{q,p}$$ 30

$${\left(\frac{\partial v}{\partial q}\right)}_{s,p}={\left(\frac{\partial \phi }{\partial p}\right)}_{s,q}$$ 31

For an equation of state containing electrical and mechanical variables, the Maxwell relationships will produce pure mechanical relationships as well as electro-mechanical ones. Equation 29 is a purely mechanical relationship readily available in archival literature.^1^ Equations 30 and 31, however, are electro-mechanical relationships that are not readily available. The three-variable equation version of Gibbs free energy is generated by Legendre transforming on the enthalpy shown in eq 28, but the transform is performed on the first term involving entropy and temperature:

dg=−sdT+vdp+ϕdq 32

The first mixed partial with the second term yields a purely mechanical relationship:

$$-{\left(\frac{\partial s}{\partial p}\right)}_{T,q}={\left(\frac{\partial v}{\partial T}\right)}_{p,q}$$ 33

The second mixed partial between the first and third terms yields an electro-mechanical one:

$${\left(\frac{\partial s}{\partial q}\right)}_{T,p}=-{\left(\frac{\partial \phi }{\partial T}\right)}_{q,p}$$ 34

The relationship of entropy with respect to charge, q, at constant temperature and pressure, is the same as voltage with respect to temperature that was obtained in two-variable thermodynamics but through specific assumptions. The third mixed partial between the second and third terms yields another electro-mechanical relationship:

$${\left(\frac{\partial v}{\partial q}\right)}_{T,p}={\left(\frac{\partial \phi }{\partial p}\right)}_{T,q}$$ 35

The Gibbs free energy, eq 32, includes three independent variables: temperature, pressure, and charge. The partial derivatives can be calculated with respect to each of these variables; thus,

$${\left(\frac{\partial g}{\partial T}\right)}_{p,q}=-S$$ 36

$${\left(\frac{\partial g}{\partial p}\right)}_{T,q}=v$$ 37

$${\left(\frac{\partial g}{\partial q}\right)}_{T,p}=\phi$$ 38

As seen in eq 37 the change in Gibbs free energy with respect to pressure at a constant temperature and charge yields volume, as shown in eq 16 for a simpler 2-variable system. The change in Gibbs free energy with respect to charge, eq 38, is equal to the Gibbs relationship of voltage, as shown in eq 11, also for the simpler 2-variable system. The next equation that will be handled here is the Helmholtz equation, generated by performing the Legendre transform on the internal energy, eq 20; thus:

dA=−sdT−pdv+ϕdq 39

The first mixed partial pressure is a purely mechanical relationship of thermodynamic properties, the same relationship obtained with a two-variable analysis on a simple compressible substance:

$${\left(\frac{\partial s}{\partial v}\right)}_{T,q}={\left(\frac{\partial p}{\partial T}\right)}_{v,q}$$ 40

As in the other cases, the second mixed partial is a combination of electrical and mechanical properties:

$$-{\left(\frac{\partial s}{\partial q}\right)}_{T,v}={\left(\frac{\partial \phi }{\partial T}\right)}_{v,q}$$ 41

The last mixed partial case is also a combination of electrical and mechanical properties:

$$-{\left(\frac{\partial p}{\partial q}\right)}_{T,v}={\left(\frac{\partial \phi }{\partial v}\right)}_{T,q}$$ 42

Relationships between entropy (a nonmeasurable property) and measurable variables are needed and these relationships are obtained through Maxwell relationships. A new internal energy equation is defined in terms of measurable variables (temperature, volume and charge). Writing the equation of state in terms of temperature, volume and charge by using the definition of an exact differential yields

$$du={\left(\frac{\partial u}{\partial T}\right)}_{v,q}dT+{\left(\frac{\partial u}{\partial v}\right)}_{T,q}dv+{\left(\frac{\partial u}{\partial q}\right)}_{T,v}dq$$ 43

The equivalent equation of state to eq 20, shown in eq 43, contains partial derivatives that are unknown, and these partial derivatives must be found. Equation 43 is then set equal to eq 20 yielding:

$$du=Tds-pdv+\phi dq={\left(\frac{\partial u}{\partial T}\right)}_{v,q}dT+{\left(\frac{\partial u}{\partial v}\right)}_{T,q}dv+{\left(\frac{\partial u}{\partial q}\right)}_{T,v}dq$$ 44

To evaluate the partial derivatives, entropy equations are needed because it is the only unmeasurable variable. Solving eq 44 for differential entropy, ds:

$$ds=\frac{1}{T}{\left(\frac{\partial u}{\partial T}\right)}_{v,q}dT+\frac{1}{T}\left[{\left(\frac{\partial u}{\partial v}\right)}_{T,q}+p\right]dv+\frac{1}{T}\left[{\left(\frac{\partial u}{\partial q}\right)}_{T,v}-\phi \right]dq$$ 45

The equation for entropy contains three unknown variables: the partial derivative of internal energy with respect to temperature, with respect to specific volume, and with respect to charge, thus using the definition of an exact differential:

$$ds={\left(\frac{\partial s}{\partial T}\right)}_{v,q}dT+{\left(\frac{\partial s}{\partial v}\right)}_{T,q}dv+{\left(\frac{\partial s}{\partial q}\right)}_{T,v}$$ 46

Equation 46 can now be used to solve for the unknown partial derivatives by setting it equal to second equation of state shown in eq 45. Maxwell relationships are then used to relate the partial derivatives to measurable properties as shown in eqs 25–27, 29–31, 33–35, and 40–42. The equation of state for internal energy, by substituting the relevant terms, is

$$du={\left(\frac{\partial u}{\partial s}\right)}_{v,q}ds+{\left(\frac{\partial u}{\partial v}\right)}_{s,q}dv+{\left(\frac{\partial u}{\partial q}\right)}_{s,v}dq$$ 47

If eq 47 is now set equal to eq 20, the partial derivatives can be replaced with thermodynamic properties:

$${\left(\frac{\partial u}{\partial s}\right)}_{v,q}=T$$ 48

$${\left(\frac{\partial u}{\partial v}\right)}_{s,q}=-p$$ 49

$${\left(\frac{\partial u}{\partial q}\right)}_{s,v}=\phi$$ 50

If the chain rule is applied using eq 48, the entropy partial derivative can be written as a function of temperature:

$$\left(\frac{\partial s}{\partial T}\right)=\left(\frac{\partial s}{\partial u}\right)\left(\frac{\partial u}{\partial T}\right)=\left(\frac{1}{T}\right)\frac{\partial u}{\partial T}=\frac{c_{v}}{T}$$ 51

The partial of entropy with respect to volume term in eq 46 likewise needs to be replaced with measurable thermodynamic properties, obtained from the eq 40. And the partial of entropy with respect to charge in eq 46 from [Disp-formula eq51]. So, in the end, if eqs 40, 41 and 51 are substituted into eq 46, the entropy equation of state can be rewritten in terms of measurable variables as shown:

$$ds=\frac{c_{V}}{T}dT+{\left(\frac{\partial p}{\partial T}\right)}_{v,q}dv-{\left(\frac{\partial \phi }{\partial T}\right)}_{q,v}dq$$ 52

If eq 52 is set equal to eq 46, the unknown partial derivatives can be solved for, and with the solved partials the internal energy equation is simplified as shown:

$$du=c_{v}dT+\left(T{\left(\frac{\partial p}{\partial T}\right)}_{v,q}-p\right)dv+\left(-T{\left(\frac{\partial \phi }{\partial T}\right)}_{q,v}+\phi \right)dq$$ 53

Equation 53 sets up the internal energy for reduction to practical relationships for specific circumstances (for example incompressible substances, ideal gases, etc., but not limited to these). Even keeping the specific heat as (∂u/∂T) would keep the equation general and applicable to many substances. In any case, secondary equations are also needed and come from secondary differential equations of state, like enthalpy. As shown in eq 28, the enthalpy equation of state contains nonmeasurable properties like entropy, so an equivalent equation of enthalpy is needed as a function of measurable quantities such temperature, pressure and charge. As in eq 28, the partial derivatives can be related to thermodynamic properties, and a new equation of state for enthalpy is thus defined with measurable independent variables being temperature, pressure and charge by again utilizing the definition of an exact differential:

$$dh={\left(\frac{\partial h}{\partial T}\right)}_{p,q}dT+{\left(\frac{\partial h}{\partial p}\right)}_{T,q}dp+{\left(\frac{\partial h}{\partial q}\right)}_{T,p}dq$$ 54

This equation of state must equal (28); thus,

$$Tds+vdp+\phi dq={\left(\frac{\partial h}{\partial T}\right)}_{p,q}dT+{\left(\frac{\partial h}{\partial p}\right)}_{T,q}dp+{\left(\frac{\partial h}{\partial q}\right)}_{T,p}dq$$ 55

Solving for ds,

$$ds=\frac{1}{T}{\left(\frac{\partial h}{\partial T}\right)}_{p,q}dT+\frac{1}{T}\left[{\left(\frac{\partial h}{\partial p}\right)}_{T,q}-v\right]dp+\frac{1}{T}\left[{\left(\frac{\partial h}{\partial q}\right)}_{T,p}-\phi \right]dq$$ 56

The exact differential for entropy as a function of temperature, pressure and charge, as indicated by eq 56, is also:

$$ds={\left(\frac{\partial s}{\partial T}\right)}_{p,q}dT+{\left(\frac{\partial s}{\partial p}\right)}_{T,q}dp+{\left(\frac{\partial s}{\partial q}\right)}_{T,p}dq$$ 57

Since eq 28 indicates enthalpy to be a function of entropy, pressure and charge, it then follows that the exact differential by definition is

$$dh={\left(\frac{\partial h}{\partial s}\right)}_{p,q}ds+{\left(\frac{\partial h}{\partial p}\right)}_{s,q}dp++{\left(\frac{\partial h}{\partial q}\right)}_{s,v}dq$$ 58

Setting eq 58 equal to (28) results in

$${\left(\frac{\partial h}{\partial s}\right)}_{p,q}=T$$ 59

$${\left(\frac{\partial h}{\partial p}\right)}_{s,q}=v$$ 60

$${\left(\frac{\partial h}{\partial q}\right)}_{s,p}=\phi$$ 61

The chain rule is used here to couple entropy to temperature. If eq 59 is used with the first term in eq 58, a relationship between the change in entropy with respect to temperature can be achieved as shown:

$$\frac{\partial s}{\partial T}=\left(\frac{\partial s}{\partial h}\right)\left(\frac{\partial h}{\partial T}\right)=\frac{c_{p}}{T}$$ 62

For the second term in eq 57, the mixed partial obtained from the Gibbs free energy relationship show how the change in entropy with respect to pressure is equal to the change in volume with respect to temperature, as shown in eq 33. Also, the last term is obtained from the Gibbs free energy equation where the change in entropy with respect to charge is equal to the change in voltage with respect to temperature, (34). Thus,

$$ds=\frac{c_{p}}{T}dT-{\left(\frac{\partial v}{\partial T}\right)}_{T,q}dp-{\left(\frac{\partial \phi }{\partial T}\right)}_{q,p}dq$$ 63

Equating eq 63 with eq 56, a new enthalpy equation of state is obtained in terms of measurable properties:

$$dh=c_{p}dT+\left(-T{\left(\frac{\partial v}{\partial T}\right)}_{p,q}+v\right)dp+\left(-T{\left(\frac{\partial \phi }{\partial T}\right)}_{p,q}+\phi \right)dq$$ 64

As with eq 53, leaving the specific heat as ∂h/∂T would keep the equation even more general as it is already applicable to many different substances. Equations 53 and 64 are more comprehensive equations for nonmeasurable properties in terms of measurable ones. More comprehensive, at least, from 2-variable ones in many textbooks that are reduced cases of these.^1^ What matters at this point then is how to apply these equations, or at least how to reduce these to model PEM fuel cell systems.

## Results

The internal energy and enthalpy equations of state, shown in eqs 53 and 64, consist of three measurable independent variables. Initial insight gained by doing a more comprehensive 3-variable thermodynamic analysis includes the possibility of energy changing into voltage or heat generation that will increase temperature, both mechanical and electrical directly observable in the equations. The new equations of state also allow for simplifications to be made, such as assuming an ideal gas, that will reduce them to even simpler terms. As will be shown, this will have the effect of reducing these comprehensive differential equations into simpler, special-case ones derived for two-variable systems. The three-variable solution also produces a new Gibbs free energy equation along with a new Helmholtz equation shown in eqs 32 and 39, respectively. But in addition to the conventional two-variable end results, there are four additional equations of state, yet be named, obtained from the three-variable equation set as will be shown here. As was stated earlier, this produces new thermodynamic relationships between the properties. In the case of two variable systems, four equations are obtained (2^2^), but in the case of three variables the yield is eight (2^3^). The first new equation of state is referred to here as f, obtained by performing the Legendre transform on eq 20 changing the last term in terms of charge and voltage:

df=Tds−pdv−qdϕ 65

The same superposition procedure as stated in the theory section of this paper is applied to Equation 65 resulting in the following set:

$${\left(\frac{\partial T}{\partial v}\right)}_{s,\phi }={\left(\frac{\partial p}{\partial s}\right)}_{v,\phi }$$ 66

$${\left(\frac{\partial T}{\partial \phi }\right)}_{s,v}=-{\left(\frac{\partial q}{\partial s}\right)}_{v,\phi }$$ 67

$${\left(\frac{\partial p}{\partial \phi }\right)}_{s,v}=-{\left(\frac{\partial q}{\partial s}\right)}_{v,\phi }$$ 68

Performing the Legendre transform on Gibbs equation, 32, yields a new function j; thus,

dj=−sdT+vdp−qdϕ 69

The Maxwell relationships are then:

$$-{\left(\frac{\partial s}{\partial p}\right)}_{T,\phi }={\left(\frac{\partial v}{\partial T}\right)}_{p,\phi }$$ 70

$${\left(\frac{\partial s}{\partial \phi }\right)}_{T,p}={\left(\frac{\partial q}{\partial T}\right)}_{p,\phi }$$ 71

$${\left(\frac{\partial v}{\partial p}\right)}_{T,\phi }=-{\left(\frac{\partial q}{\partial T}\right)}_{p,\phi }$$ 72

Similar to the other cases there are properties that couple mechanical and electric properties. The third new unnamed equation obtained is based on the Legendre transform of the enthalpy, h, eq 28. As with the last two transforms, the last term contains voltage and current:

dl=Tds+vdp−qdϕ 73

And the resulting thermodynamic relationships here are

$${\left(\frac{\partial T}{\partial p}\right)}_{s,\phi }={\left(\frac{\partial v}{\partial s}\right)}_{p,\phi }$$ 74

$${\left(\frac{\partial T}{\partial \phi }\right)}_{s,p}=-{\left(\frac{\partial q}{\partial s}\right)}_{p,\phi }$$ 75

$${\left(\frac{\partial v}{\partial \phi }\right)}_{s,p}=-{\left(\frac{\partial q}{\partial p}\right)}_{s,\phi }$$ 76

Similar to the other cases, these are mixed electrical and mechanical properties. The final equation, and corresponding equality set, are obtained from the Legendre transform of the Helmholtz function, eq 39:

dk=−sdT−pdv−qdϕ 77

Resulting in

$$-{\left(\frac{\partial s}{\partial v}\right)}_{T,\phi }=-{\left(\frac{\partial p}{\partial T}\right)}_{v,\phi }$$ 78

$$-{\left(\frac{\partial s}{\partial \phi }\right)}_{v,T}=-{\left(\frac{\partial q}{\partial T}\right)}_{v,\phi }$$ 79

$$-{\left(\frac{\partial p}{\partial \phi }\right)}_{T,v}=-{\left(\frac{\partial q}{\partial v}\right)}_{T,\phi }$$ 80

As in the other three variable cases, the mixed partials from eq 77 will produce relationships between mechanical properties, as shown in eq 78, electrical relationships with temperature, as shown in eq 79, and electro-mechanical relationships as shown in eq 80. Equation 79 could be used to relate the change in entropy with respect to voltage to a measurable property. These numerous relationships are useful in combined electromechanical systems such as PEM fuel cells. The point here is that more information comes out of an analysis with 3-variables than with multiple iterations of simpler 2-variable systems, some of these relationships not obvious from the onset of analysis.

## Discussion

In reference to electrochemical systems utilizing two variables^1,2–6^ , Weber et al, Ramousse et al.,^10^ Das et al.^11^ and Shi^12^ note that the theoretical voltage of the PEM fuel cell is dependent on the Gibb’s free energy. This relationship is also obtained here in the three-variable system considered. The theoretical voltage of the PEM fuel cell depends on only pressure and temperature, but assumptions were made regarding both to simply the Gibbs equation as shown in eq 7 and ([Disp-formula eq10]):

$$\phi =\frac{\Delta G}{nF}$$ 81

The two variable theoretical voltage, eq 81, is derived from a simple mechanical substance under constant pressure and constant temperature conditions. The same result was achieved here with the three variable analysis as shown in eq 38, confirming in this instance (one of many) that the three-variable equation of state simplifies to the two, and validates the current methodology. The change in Gibbs free energy/electrical for a two-variable thermodynamics system is derived from a simple mechanical substance under isothermal and isobaric conditions, this approach approximates the electro-mechanical relationship shown in eqs 14 and 18. These electro-mechanical relationships are obtained unsurprisingly with the three-variable method, eqs 34 and 41.

The reference temperature of chemical and electrochemical reactions is at 25 °C (298.15K), as a result the voltage and enthalpy change when the temperature of the system varies from that reference temperature. And when the temperature changes, theoretical voltage will also change, and the voltage then needs correction. Abdin et al.^13^ noted that change in voltage with respect to temperature is the same as the change in entropy with respect to charge. There is a relationship between charge and the number of moles of a substance, where Faraday’s law can be used to equate them. As eqs 34 and 41 show here, the three-variable relationship of entropy with respect to charge equates to voltage change with respect to temperature. The key difference between the eq 34 and 41 is that one of the independent variables is at a constant pressure while the other at constant volume (current fuel cell models assume that pressure is constant). Abdin et al.^13^ used the entropy change with respect to charge to correct the open circuit voltage:

$$\phi_{rev}=\phi_{rev}^{0}+\left(T-T_{ref}\right)\frac{\Delta S}{nF}$$ 82

In addition, they used eq 15 to compute the change in voltage with respect to temperature. It should be noted that the change in entropy with respect to charge is also equal to the change in voltage with respect to temperature as shown in eq 34 which originates from the three-variable analysis. Abdin et al.^13^ noted that the change in entropy with respect to charge, $\left(\frac{\Delta S}{\mathrm{nF}}\right)$ , is equal to −0.9 mV/°C. They also referred to the work of Amphlett et al.,^14^ where the change in voltage comes from the change in entropy with respect to charge:

$$\frac{\Delta S}{nF}=-0.85x10^{-3}\frac{V}{K}$$ 83

Amphlett et al.^14^ did not directly state that the change of entropy with respect to charge is equal to the change in voltage with respect to temperature, eq 34, nor how a value of −0.9 mV/°C was obtained. Bernardi et al.^15^ noted that the reversable voltage depends on pressure and temperature:

$$\frac{\Delta S}{nF}=-0.85\times 10^{-3}\frac{V}{K}$$ 84

One common term between Abdin et al.^13^ and Bernardi et al.^15^ is −0.9×10−3(VC°)(T−298) , which signifies the reversible change of voltage with respect to temperature. The origin of the 0.9 mV/K was not explicitly given but the change in entropy with respect to charge will be calculated and its value will be compared to the reference value. Another model was developed by Weber and Newman^16^ where the enthalpy change is related to the voltage change with respect to temperature, noting that the reversable voltage has a nonentropy-based term:

$$\phi_{H}=\frac{\Delta h}{2F}=\phi^{o}-T\left(\frac{d\phi }{dT}\right)$$ 85

If the three variable enthalpy equation, 54, is under constant pressure and temperature, the first and second term will become zero, and will simplify to eq 85. For this to be true, the entropy change with respect to charge must equal the change in voltage with respect to temperature; thus,

$$\frac{\partial s}{\partial q}=\frac{\Delta s}{nF}=-\frac{\partial \phi }{\partial T}$$ 86

This relationship is also obtained from one of the three variable Gibbs relationships. Equating partial derivatives shown is eq 34 produces the same result as eq 36 , which confirms the change in voltage with respect to temperature is the same as the change in entropy with respect to charge. As shown in the three variable cases in this paper, the entropy with respect to charge relationship is the same as the change in voltage with respect to charge, as shown in eqs 34 and 35, the three variable internal energy and enthalpy, as shown in eqs 53 and 64, can be used to model PEM fuel cells. The three variable enthalpy eq 64, can be simplified to eq 85 by assuming a constant temperature and pressure, and using Faraday’s law to equate charge to amount of substance. The new three variable equations are then validated against a few more fuel cell models. Webber and Newman,^16^ Ju et al.,^17^ and Chao-Yang Wang^18^ modeled PEM fuel cells, and one of the equations that was contained in all their models is the voltage change in the fuel cells with respect to temperature:

$$\phi =\phi_{0}-T\frac{\partial \phi }{\partial T}$$ 87

As previously stated, the change in voltage with respect to temperature does appear in the three-variable internal energy and enthalpy, eqs 53 and 64. Further, Bhaiya^19^ noted a Peltier effect in an electrochemical reaction:

$$\Pi =\frac{T\Delta s}{nF}$$ 88

Here, the Peltier effect shows that the voltage loss from temperature is related to the change in entropy with respect to charge. Through thermodynamic relationships between partial derivatives and thermodynamics properties, the change in entropy with respect to charge can be equated to the change in voltage with respect to temperature. In addition, the last term in eq 87 appears in the three-variable internal energy and enthalpy equations, 53 and 64, respectively. The entropy change can also be related to voltage change as shown in eqs 34 and 41. As it is not obvious, Abdin et al.^13^ and Bhaiya^19^ could not foresee that the entropy change with respect to charge is the same as voltage change with respect to temperature, something that naturally ‘falls out’ of the 3-variable analysis shown here. Weber and Newman’s^16^ PEM fuel cell voltage model is similar to Abdin’s et al.^13^ and Bhaiya’s,^19^ but there is a term relating voltage change to temperature. Weber and Newman stated the enthalpy change is related to the voltage change with respect to temperature:^16^

$$\phi_{H}=\frac{\Delta h}{2F}=\phi^{o}-T\left(\frac{d\phi }{dT}\right)$$ 89

The three variable enthalpy equation reflects this. If constant pressure and temperature are assumed, then eq 64 will be the same as eq 89. Wang and Wang^20^ pointed out a term in their fuel cell model where the heat generation is based upon the product of current with the sum of the voltage and change in voltage with respect to temperature; thus,

$$S_{T}=j\left(\phi +T\frac{d\phi }{dT}\right)$$ 90

The change in voltage with respect to temperature is shown in the last three variable internal energy equation, 53. You and Liu^21^ used the following equation as the model for computing the theoretical voltage from the PEM fuel cell:

$$\phi_{Rev}=1.23V-0.9x10^{-3}\left(T-298\right)V+\frac{RT}{4F}\ln\left(\frac{p_{H2}^{2}p_{O2}}{p_{H20}}\right)V$$ 91

The 1.23 V is the theoretical voltage of the PEM fuel cell at 25 °C, while the second term with 0.9 mV/K is the voltage loss due to temperature. Spiegel et al.^3^ modeled the reversible voltage and established a coupling between the voltage change with respect to temperature to entropy changes:

$$\phi =\phi^{o}-\frac{d\phi }{dT}\left(T-25^{{}^{\circ}}C\right)=\phi^{o}-\frac{\Delta s}{nF}\left(T-25^{{}^{\circ}}C\right)$$ 92

They noted that the change in entropy with respect to charge is the same as the change in voltage with respect to temperature. Here, eq 92 can be obtained from the three variable internal energy, eq 53, if temperature and pressure are constant. Equation 92 can also be derived from the three-variable enthalpy, eq 64, if the temperature and pressure are constant. Eikerling and Kulikovsky^22^ and Afshari and Jazayeri^23^ also expressed the model for cell voltage where the change in entropy with respect to charge is equal to −0.85 mV/°C:

$$\phi =\phi^{o}+\frac{\Delta s}{nF}\left(T-T_{o}\right)$$ 93

In the case shown, the three-variable thermodynamic analysis here shows that the change in entropy with respect to charge is equal to the change in voltage with respect to temperature as shown in eqs 34 and 41. Haghayegh et al.^24^ and Penga et al.^25^ modeled the PEM fuel cell, and like Abdin et al.,^13^ the theoretical voltage needed to be calculated. Both research efforts used two variable thermodynamics for a simple substance to correct the theoretical voltage with respect to temperature and pressure:

$$\phi =\phi^{o}+\frac{\Delta s}{nF}\left(T-298\right)-\frac{RT}{nF}\ln\left(\frac{P_{H_{2}O}}{P_{H_{2}}P_{O_{2}}^{0.5}}\right)$$ 94

Haghayegh et al.^24^ and Penga et al.^25^ show that the change in entropy with respect to charge will calculate the change in voltage with respect to temperature. The impact of pressure on voltage can be obtained in this research from three-variable Maxwell relationships. Equation 35 shows that the change in voltage with respect to pressure is equal to the change of volume with respect to change. If an ideal gas is assumed and Faraday’s law is used to relate charge to moles of substance and this is substituted in, eq 35 becomes.

$$\frac{\partial \phi }{\partial p}=\frac{\partial }{\partial q}\left(\frac{qRT}{PF}\right)=\frac{RT}{FP}$$ 95

Solving this differential equation for ϕ results in

$$\phi =\frac{RT}{F}\ln\left(P\right)+c$$ 96

Equation 96 is generic and requires the stoichiometric coefficients of the chemical reaction to be considered. The three variable pressure adjustment shown in eq 96, and the two variable counterparts shown in eqs 91 and 94 have the natural log of pressure in both equations. This is further evidence that the three variable cases reduce to the two variable ones. This also indicates that the ideal gas equation is one of the assumptions made for fuel cell modeling in prior research, even if only implied. Cao et. al^26^ modeled the heat generation of the PEM fuel cell with the cathode catalysis layer (CCL) through the following equation, where the theoretical voltage of the cathode was corrected with temperature:

$$S_{T}=j\left(\phi +T\frac{d\phi }{dT}\right)+\frac{j^{2}}{\sigma_{m}}+\frac{j^{2}}{\sigma_{s}}+S_{l}h_{fg}$$ 97

Like the other cases, the source term contains the change in voltage with respect to temperature. As shown in eq 64, the three-variable change in enthalpy matches the voltage change term obtained in three-variable thermodynamic enthalpy. Yang-Weng^18^ and Ju et al.^27^ expressed the heat generation as

$$Q_{heat}=\left(\phi_{o}-\phi_{Discharge}\right)jA+jAT\left(\frac{-d\phi }{dT}\right)$$ 98

This is another case where the change in voltage with respect to temperature appears like in the three variable internal energy, eq 53, and the enthalpy eq 64. In the existing fuel cell models, developers either use entropy change with respect to charge, or the change in voltage with respect to temperature. The three variable thermodynamic analysis here explicitly shows that these variables are equivalent, as shown in eqs 34 and 41. Further, in the PEM fuel cell there are two scenarios, the first where water vapor is produced, and the second where liquid water is produced, both at the cathode:

$$H_{2}\left(g\right)+\frac{1}{2}O_{2}\left(g\right)\to H_{2}O\left(g\right)$$ 99

$$H_{2}\left(g\right)+\frac{1}{2}O_{2}\left(g\right)\to H_{2}O\left(l\right)$$ 100

The net reactions shown in these equations can also be written as an oxidation and reduction reactions. The oxidation reaction at the anode, which is not impacted by the phase of water, and the reduction reaction (impacted by the phase of water) pending if vapor or liquid water is produced are, respectively:

$$H_{2}\to 2H^{+}\left(g\right)+2e^{-}$$ 101

$$2H^{+}\left(g\right)+\left(\frac{1}{2}\right)O_{2}\left(g\right)+2e^{-}\to H_{2}O\left(g\right)$$ 102

$$2H^{+}\left(g\right)+\left(\frac{1}{2}\right)O_{2}\left(g\right)+2e^{-}\to H_{2}O\left(l\right)$$ 103

In the oxidation and reduction reaction, two electrons are transferred. The coefficients in front of the atoms/molecules are the stoichiometric coefficients. One molecule of hydrogen is required along with half of an oxygen molecule, the combination yielding one molecule of water. The quantities are obtained from eqs 99 and ([Disp-formula eq100]) and the entropy values for the substances are found from the following table [[Table tbl1]]:

**Table 1:** Entropy of the Fuel Cell Reactants and Products

substance	entropy (*s*) J/mol K
H_2_ (g)	130.57
O_2_ (g)	205.03
H_2_O (g)	188.72
H_2_O (l)	69.95

The total change in entropy is −44.365 J/(mol K) if the entropy change is divided by the change in charge (q or nF) when water vapor is produced by the fuel cell:

$$\left(\frac{\mathrm{\Delta s}}{\mathrm{\Delta q}}\right)=\frac{-130.57\frac{J}{\mathrm{mol}K}-0.5x\frac{205.03\mathrm{kJ}}{\mathrm{mol}K}+188.72\frac{J}{\mathrm{mol}K}}{2x9,6485\frac{C}{\mathrm{mol}}}=\frac{-44.365J/\left(\mathrm{mol}K\right)}{192,090\frac{C}{\mathrm{mol}}}=-2.30x10^{-4}\frac{V}{K}$$ 104

In the case of liquid water being produced, the change in voltage with temperature is computed as

$$(\frac{\mathrm{\Delta s}}{\mathrm{\Delta q}})=\frac{-130.57\frac{J}{\mathrm{mol}K}-0.5\frac{205.03J}{\mathrm{mol}K}+69.95\frac{J}{\mathrm{mol}K}}{2x9,6485\frac{C}{\mathrm{mol}}}=\frac{-163.135J/\left(\mathrm{mol}K\right)}{192,090\frac{C}{\mathrm{mol}}}=-8.45x10^{-4}\frac{V}{K}$$ 105

The value obtained by the change in entropy with respect to charge is close to the value used by Abdin et al.,^13^ Amphlett et al.,^14^ Bernardi et al.,^15^ Weber and Newman,^16^ Bhaiya,^19^ and You and Liu.^21^ This shows that the change in entropy with respect to charge is the same as the change in voltage with respect to temperature. In addition to the entropy change of the fuel cell, there is a thermo-electric effect that will contribute to the change of voltage with respect to temperature, known at the Peltier effect. Weber and Newman^28^ combined the entropy and Peltier effect together to produce a numeric value of 0.848 mV/K, which is the change in voltage with respect to temperature, and is the same value of the change in entropy with respect to charge as shown in eq 105. This value reflects the change in entropy with respect to charge in the liquid water scenario rather than the water vapor one. Bhaiya^19^ noted that the Peltier effect is based on the entropy change with respect to charge as shown in eq 88. In the two-variable system, there is the possibility of an isentropic process, so the change in entropy is set equal to zero; thus,

$$0=\frac{c_{V}}{T}dT+{\left(\frac{\partial p}{\partial T}\right)}_{v,q}dv$$ 106

In this case a relationship between temperature and volume can be achieved by setting the terms equal and solving the differential equation. With the three-variable system in this paper, the entropy equation of state—a function of three variables s per eqs 52 and 63—can be set equal zero. But here there are three independent variables instead of two so this would introduce a dual variable process. With two entropy equations and 3 variables, a total of six thermodynamic processes can be obtained. The three-variable solution detailed here takes advantage of Maxwell’s mixed partials to derive thermodynamic properties that are a mix of mechanical and electrical properties. In the instance of the three-variable enthalpy, eq 31, one of the mixed partials states that change in volume with respect to charge is equal to the change in voltage with respect to pressure, eq 35. During the derivation of the three-variable state equations, the entropy ones were obtained as shown in eqs 52 and 63. In the two-variable system, isentropic relationships are obtained between the two independent variables. In the three variable equations, eqs 52 and 63, there are three independent variables as seen when the change in entropy to zero:

$$0=\frac{c_{V}}{T}dT+{\left(\frac{\partial p}{\partial T}\right)}_{v,q}dv-{\left(\frac{\partial \phi }{\partial T}\right)}_{q,v}dq$$ 107

$$0=\frac{c_{p}}{T}dT-{\left(\frac{\partial v}{\partial T}\right)}_{T,q}dp-{\left(\frac{\partial \phi }{\partial T}\right)}_{q,p}dq$$ 108

This is essential to determine the best efficiency that can be achieved from the first and second laws of thermodynamics. If entropy change, along with another independent variable, are set to zero an isentropic and one special case (isothermal, isobaric, etc.) can be achieved. There are thus six new processes introduced, the first one being isentropic and isothermal, then isentropic and isochoric, isentropic and isocharge, isentropic and isobaric, isentropic and isothermal, and isentropic and isocharge. The new processes also allow for simplifications such as an ideal gas. In the case of a two-variable simple compressible substance^1^, the differential entropy is set to zero, so the relationship between the two remaining variables can be obtained by solving the differential equation. But here with the three-variable system additional relationships are obtained. The first case is that of an isentropic and isothermal process obtained from eq 107 by also setting dT = 0:

$$0={\left(\frac{\partial p}{\partial T}\right)}_{v,q}dv-{\left(\frac{\partial \phi }{\partial T}\right)}_{q,v}dq$$ 109

The second case is an isentropic and isochoric process obtained from eq 107 by setting dv = 0:

$$0=\frac{c_{V}}{T}dT-{\left(\frac{\partial \phi }{\partial T}\right)}_{q,v}dq$$ 110

The third is an isentropic and isocharge process that yields the same result as from the conventional two-variable simple compressible substance by setting dq = 0:

$$0=\frac{c_{V}}{T}dT+{\left(\frac{\partial p}{\partial T}\right)}_{v,q}dv$$ 111

From the second entropy equation shown in eq 108, three more special cases can be obtained from equations:

$$0=\frac{c_{p}}{T}dT-{\left(\frac{\partial \phi }{\partial T}\right)}_{q,p}dq$$ 112

$$0=-{\left(\frac{\partial v}{\partial T}\right)}_{T,q}dp-{\left(\frac{\partial \phi }{\partial T}\right)}_{q,p}dq$$ 113

$$0=\frac{c_{p}}{T}dT-{\left(\frac{\partial v}{\partial T}\right)}_{T,q}dp$$ 114

Faraday’s law will also be used to relate the change in charge to changes in moles:

q=nF 115

The ideal gas equation can be rearranged in terms of pressure:

$$p=\frac{RT}{v}$$ 116

The partial derivative of the ideal gas equation can then be taken with respect to temperature:

$${\left(\frac{\partial p}{\partial T}\right)}_{v}=\frac{R}{v}$$ 117

The change in voltage with respect to temperature is taken from eq 105, and if the value from eqs 105 and 117are substituted into eqs 109 and 115 is used to replace charge with moles, the following is obtained:

$${\left(\frac{v_{2}}{v_{1}}\right)}^{R}=e^{-0.845\times 10^{-3}V/K\left(\left(q_{2}-q_{1}\right)\right)}$$ 118

The new relationship indicates how the change in charge will impact volume. In the case of eq 112, which is an isentropic and isochoric case, the change in voltage with respect temperature is taken from eq 105 and substituted into (112) along with Faraday’s law, and the differential equation solved yielding

$${\left(\frac{T_{2}}{T_{1}}\right)}^{c_{v}}=e^{-0.845\times 10^{-3}V/K\left(\left(q_{2}-q_{1}\right)\right)}$$ 119

For the isentropic and constant charge case, the ideal gas equation is substituted into (111) and the differential equation is solved yielding:

$${\left(\frac{T_{1}}{T_{2}}\right)}^{1/\gamma -1}=\left(\frac{v_{2}}{v_{1}}\right)$$ 120

The isentropic and constant charge case shown in eq 120 is the same as the two-variable isentropic relationship for a simple compressible substance. The two variable case also implies constant charge. The second equation of state of entropy shown in eq 108 was further simplified to eq 112 by stating the process to be isentropic and isothermal by making ds = dT = 0. If the value from eq 105 is substituted into eq 112 along with the ideal equation and adding Faradays law, and solving this differential equation results in

$${\left(\frac{T_{2}}{T_{1}}\right)}^{C_{P}}=e^{\left(-0.845\times 10^{-3}V/K\left(F\left(n_{2}-n_{1}\right)\right)\right)}$$ 121

If the ideal gas equation is then substituted into eq 105 and the differential equation is solved, and using Faradays law,

$${\left(\frac{P_{2}}{P_{1}}\right)}^{R}=e^{(-0.845\times 10^{-3}V/K\left(q_{2}-q_{1}\right))}$$ 122

The relationship between the pressure and the number of moles indicates how the pressure of the system will change with a change in reactants. The last process here is an isentropic and constant charge process as shown in eq 114. To solve this, the ideal gas equation is substituted in for the partial derivative and the differential equation is solved resulting in

$$\left(\frac{T_{2}}{T_{1}}\right)={\left(\frac{v_{2}}{v_{1}}\right)}^{\left(\gamma -1/\gamma \right)}$$ 123

The relationship shown in eq 123 is the same relationship for a simple mechanical substance, as most readers will recognize, which indicates the three-variable equations of state will simplify to the more familiar two-variable equations of state, as should be expected. Up to this point in time, the modeling of a PEM fuel cell from voltage to heat generation is based primarily on two variable thermodynamics of a simple mechanical substance assuming a constant temperature and constant pressure to yield the presently accepted models. The goal here is to update the modeling strategy and understanding of electrochemical systems to allow for variations in pressure and temperature among several other properties. In this sense, three-variable thermodynamic state equations seem to be a better path to obtaining useful relationships, some of which are and have been common in textbooks for many years, but some that are not fully known or used yet, such as those shown in this research.

## Conclusions

The conventional method of two-variable thermodynamics published in almost all thermodynamic textbooks is based on, for instance, a simple substance with a single relevant work mode. In a two-variable mechanical system, such as with a simple compressible substance, energy can either be transformed into mechanical work or heat. To provide more understanding of electrochemical systems with both mechanical and electrical properties that tend to be more complex, a three-variable thermodynamic analysis is thus proposed here as a better starting point. This should be seen as a more comprehensive analysis getting to the same end goal of obtaining relationships for nonmeasurable properties in terms of measurable ones.

A three-variable electrical and mechanical analysis shown in this paper incorporates the effects of pressure, volume, temperature, voltage, charge, and entropy, such as would be the case in PEM fuel cell systems, as it is no longer a simple mechanical substance but rather a substance with simultaneous electrical and mechanical properties. As shown in the discussion section, reducing the 3-variable system with appropriate simplifications down to a 2-variable one results in expressions that have been proposed in a good number of prior publications in the archival literature, many of these being a combination of theory and experimental work.

For instance, the impact of temperature can be seen on internal energy, u, and enthalpy, h, as shown in eqs 53 and 64, respectively. If the three-variable enthalpy is simplified to a constant pressure and temperature, then the conventional two-variable system^1^ is obtained. In the PEM fuel cell, however, constant temperature and pressure are not generally appropriate. The proposed three-variable Gibbs energy equation, 32, produces the same results obtained from the conventional fuel cell modeling with two variables, but with additional information still at hand here. The theoretical voltage obtained from the three-variable Gibbs free energy, eq 38, is the same as the two-variable derivation, eq 10. The change in voltage with respect to temperature is also linked to the change in entropy with respect to charge, obtained from the three-variable analysis, eq 41, correlating to the current two variable system shown in eq 14. Also, the pressure effects of the three-variable analysis, eq 35, equals that of the two variable method shown in eq 18.

Another of the outcomes from the analysis shown here is that the entropy change with respect to charge is equal to the change in voltage with respect to temperature, which is one of the many three-variable results, as shown in eqs 34 and 41. This validates the current modeling done by Abdin et al.^13^, Webber and Newman^16^, Ju et al.^17^ Wang and Wang^20^, Eikerling and Kulikovsky^22^, Afshari and Jazayeri^23^, Spiegel^3^, Haghayegh et al.^24^, Penga et al.^25^, Cao et al.^26^, Yang-Weng^18^ and Ju et al.^27^. Their models, however, were all derived from two-variable thermodynamics and are special cases of that shown in the current research.

One of the advantages of the three-variable method outlined here is that it also yields four additional equations of state (yet to be named) as shown in eqs 65, 69, 73 and 77, producing new relationships between thermodynamic properties some of which are mechanical and others electrical, as seen in eq 31. These relationships are not born out of 2-variable thermodynamics published nor are they easy to foresee. The additional relationships can provide more insight into PEM fuel cell operation and improve modeling. With the new method proposed here, conventional assumptions can still be made, for instance a real gas or the Van derWaal equation could be used instead of the ideal gas equation. In general, two-variable thermodynamic relationships can be obtained from simplifying the three variable results, but the latter gives way to additional relationships to yet consider, a consideration of future research.

## Nomenclature

A: Helmholtz energy (kJ/kg)
A: area (m^2^)
F: Faraday’s constant (C/mol)
H: magnetic field strength (A/m)
M: magnetic moment (H/m)
N: component moles (moles)
P: dipole moment (C m)
S: entropy (kJ/C)
S_l_: liquid quality (kJ/C)
S_T_: energy source (W/m^2^)
W_e_: electrical work (W)
g: Gibbs free energy (kJ/kg)
h: enthalpy (kJ/kg)
p: pressure (kPa)
s: entropy (kJ/kg°C)
T: temperature (°C)
u: internal energy (kJ/kg°C)
v: specific volume (m^3^)
W_e_: electrical work (J)
*g*: Gibbs free energy (kJ/kg)
h_fg_: heat of vaporization (kJ/kg)
ϕ: voltage (V)
j: current density (A/m^2^)
*n*: amount of substance (moles)
p_H2_: pressure of hydrogen (kPa)
p_O2_: pressure of oxygen (kPa)
p_H2O_: pressure of water (kPa)
*q*: charge (C/mol)
T: absolute temperature (K)
Π: Peltier coefficient (V/°*C*)
ϵ: electric field (V/C)
ϵ_*m*_: strain (m/m)
σ_m_: conductance of the electrolyte (S/m)
σ_s_: conductance of the membrane (S/m)
σ: stress (N/m^2^)
μ: chemical potential (kJ/kg)
ϕ°: voltage at 25 °C or 298 K (V)
